# The effect of beliefs about alcohol’s acute effects on alcohol priming and alcohol-induced impairments of inhibitory control

**DOI:** 10.1371/journal.pone.0201042

**Published:** 2018-07-26

**Authors:** Graeme Knibb, Carl. A. Roberts, Eric Robinson, Abi Rose, Paul Christiansen

**Affiliations:** Department of Psychological Sciences, University of Liverpool, Liverpool, United Kingdom; Univerity of Salzburg, AUSTRIA

## Abstract

Acute alcohol administration can lead to a loss of control over drinking. Several models argue that this ‘alcohol priming effect’ is mediated by the effect of alcohol on inhibitory control. Alternatively, beliefs about how alcohol affects behavioural regulation may also underlie alcohol priming and alcohol-induced inhibitory impairments. Here two studies examine the extent to which the alcohol priming effect and inhibitory impairments are moderated by beliefs regarding the effects of alcohol on the ability to control behaviour. In study 1, following a priming drink (placebo or .5g/kg of alcohol), participants were provided with bogus feedback regarding their performance on a measure of inhibitory control (stop-signal task; SST) suggesting that they had high or average self-control. However, the bogus feedback manipulation was not successful. In study 2, before a SST, participants were exposed to a neutral or experimental message suggesting acute doses of alcohol *reduce* the urge to drink and consumed a priming drink and this manipulation was successful. In both studies craving was assessed throughout and a bogus taste test which measured *ad libitum* drinking was completed. Results suggest no effect of beliefs on craving or *ad lib* consumption within either study. However, within study 2, participants exposed to the experimental message displayed evidence of alcohol-induced impairments of inhibitory control, while those exposed to the neutral message did not. These findings do not suggest beliefs about the effects of alcohol moderate the alcohol priming effect but do suggest beliefs may, in part, underlie the effect of alcohol on inhibitory control.

## Introduction

Acute alcohol administration can lead to a loss of control over drinking, a process known as the alcohol priming effect [1]], characterised by increased objective (e.g. *ad libitum* alcohol consumption) and subjective (e.g. craving) measures of alcohol-seeking [2–5]]. It has been suggested that the alcohol priming effect is mediated by alcohol-induced impairments in inhibitory control [6]]. However, although previous research has reliably demonstrated inhibitory control to be impaired following consumption of moderate (.40-.65g/kg) doses of alcohol [7–10]]; little research shows that such impairments mediate the alcohol priming effect.

Presently, only one study provides evidence that alcohol-induced impairments of inhibitory control correlate with subsequent alcohol consumption [11]]. This study found that alcohol-induced impairments following a moderate (.65g/kg) priming dose of alcohol were correlated with *ad lib* alcohol consumption measured in a subsequent testing session. Problematically, this does not offer convincing evidence that this impairment underlies the alcohol priming effect as participants did not consume a priming dose of alcohol in the same session as their alcohol-seeking was assessed. Critically, there is no evidence that alcohol-induced inhibitory impairments can account for *ad lib* alcohol consumption when measured in the same testing session [4, 12–15]].

Moreover, there is a growing evidence base suggesting that the alcohol priming effect cannot be wholly attributable to the pharmacological effects of alcohol on cognitive processes as anticipated (placebo) effects are also important. For example, consumption of placebo alcohol has been found to promote craving and increase *ad lib* alcohol consumption [4, 16–18]]. This anticipated effect is not limited to alcohol priming; placebo alcohol has also been found to impair inhibitory control [18]], motor performance [19–21]] and increase automatic approach tendencies [4]]. Furthermore, anticipated effects of alcohol may be, at least in part, dependent on individual differences in alcohol-outcome expectancies. For example, impairments in inhibitory control and motor performance following placebo-alcohol are correlated with expectation of alcohol-induced cognitive impairment [18–21]]. Moreover, when participants were explicitly led to expect alcohol-induced impairment (but unknowingly consume placebo alcohol) their performance on a pursuit rotor task was improved relative to participants who were led to believe their performance would be enhanced, as participants in the former condition attempted to compensate for expected impairments [20]].

Beliefs about one’s ability to control behaviour (regardless of actual ability) are likely to be important in explaining self-regulation. For example, participants led to believe that they possess high levels of ‘willpower’ or have ‘self-control resources’ available to them have been found to better regulate their behaviour than those led to believe they lack willpower/self-control [22, 23]]. Critically, similar findings have also been demonstrated with regard to controlling substance intake.

Nordgren and colleagues [24]] provided smokers with bogus feedback, following a cognitive task, leading them to believe they had either high or low levels of ‘impulse-control’; the authors report that those led to believe they possessed high impulse control were significantly more likely to smoke (notably this finding was actually non-significant, p = .06). However, Jones et al [[25]] led social drinkers to believe that they had either high or low levels of restraint prior to an *ad lib* alcohol consumption session. More alcohol was consumed by participants led to believe that their ability to control their behaviour was high. Problematically, neither of the aforementioned studies contained an average control group (i.e. a group told that their ability to control their behaviour was average) so it is unclear whether group differences in substance use were the product of the belief that ability to control behaviour was high or low.

Taken together, evidence suggests that manipulating beliefs about the ability to self-regulate is likely to influence *ad lib* alcohol consumption. It also suggests that the alcohol priming effect and alcohol-induced inhibitory control impairments are, at least in part, the product of the belief that alcohol has been consumed and will impair self-regulation. The present research aims to assess the hypothesis that the alcohol priming effect, and alcohol-induced impairments of inhibitory control, is influenced by the belief that alcohol can impair behavioural control.

Two studies were conducted; both consisted of two experimental sessions with participants receiving a priming dose of alcohol (.5g/kg) in one session and a placebo in the other. In both studies participants completed measures of craving and subjective intoxication at three time points (baseline, post-drink, and end of session), a measure of inhibitory control following the priming drink (stop signal task; SST), and a bogus taste task at the end of each session. In the first study participants were told that their performance on the SST was indicative of their ability to control their behaviour following alcohol. They were provided with bogus feedback following the task and were led to believe that they had high or average self-control similar to previous work [24, 25]]. An ‘average-control’ condition was used so that the direction of the effect of high self-control beliefs could be properly elucidated.

In the second study, rather than implying that alcohol may lead to impaired self-regulation following alcohol (as with study 1), participants were explicitly told that consuming a small dose of alcohol *reduces* the urge to drink (experimental condition) or were provided with a neutral control message. Given that alcohol-related cues may impair inhibitory control and increase craving [26]] and bar-like environments may increase *ad lib* drinking [27, 28]] we conducted both studies in a semi-naturalistic bar laboratory.

Research has shown that if an individual believes that their self-regulate is poor they show poorer self-regulation [22, 23]], but the direction of this effect on substance use has not been properly elucidated [24, 25]]. Therefore, for the first study, it was hypothesised that the alcohol priming effect would be mitigated in participants led to believe that their ability to control their behaviour was high. So that participants within the high-control condition would consume less alcohol in the bogus taste task and report lower post-manipulation levels of craving than those within the average-control condition. For the second study it was also predicted the alcohol priming effect would be mitigated among participants led to believe that a small dose of alcohol would reduce their urge to drink (experimental condition) relative to the control condition. Finally, for study 2, it was hypothesised that alcohol-induced impairments of inhibitory control would be reduced within this group as demonstrated by improved performance on the SST relative to the control condition.

## Study 1: Method

### Participants

Eighty-one participants (44 male, 37 female) aged 18–49 (mean age 23.98 *±* 6.49) were recruited via advertisements placed around the University of Liverpool or in return for course credit. The sample sizes for both studies were determined by a power calculation, using GPower [29]] for detecting an effect of manipulated beliefs about self-regulation on *ad lib* alcohol consumption (η_p_^2^ = .08, based on Jones et al [25]]). According to the power analysis the target sample size for 80% power was *N* = 70 we recruited more than this to account for potential removal of outliers. Participants were required to drink at least 10 UK units (1 UK unit = 8g alcohol) in an average week, be fluent English speakers, and like the taste of beer. Exclusion criteria included a past or present alcohol disorder, being on medication which may be affected by alcohol and current illness which may increase alcohol sensitivity. Females who were currently pregnant or breastfeeding were also excluded. The study was ethically approved by the University of Liverpool Research Ethics Committee and all participants in both studies provided written informed consent.

### Design

This study used a mixed design with a within-subject factor of drink (alcohol/placebo) and a between-subject factor of condition (average-control group and high-control group). Participants attended the laboratory twice with at least 48hrs between sessions. During these sessions they consumed a placebo or an alcoholic drink in a counterbalanced order. Participants were allocated to either experimental or control conditions using fixed block allocation (AABB). Drink content was single blinded.

### Materials

#### Drinks preparation

The dose of alcohol participants received was calculated at .5g per kg of body weight. The alcohol drink contained vodka (Co-op Imperial Vodka, 37.5% alcohol by volume; ABV) which was mixed with chilled lemonade in the ratio of one part vodka to three parts lemonade. The placebo drink consisted of lemonade (Co-op Sparkling Lemonade) of an identical total volume to the alcoholic drink. For both drinks an atomiser was used to spray vodka mist on the surface of the drink and the rim of the glass [18]. This procedure and dose was used for both studies.

#### Alcohol use disorders identification test (AUDIT)

The AUDIT [30]] was used to assess patterns of harmful drinking. The AUDIT is comprised of 10 items and is scored out of a possible 40. Scores over 8 are indicative of hazardous or harmful drinking patterns. (Study 1 α = .75; Study 2 α = .66).

#### Time line Follow Back (TLFB)

TLFB questionnaire is a self-report measure of alcohol consumption [31]] Participants were required to retrospectively record the amount of alcohol units (1 UK unit = 8g of alcohol) they had consumed over the two weeks prior to the first experimental session.

#### Leeds dependence questionnaire (LDQ)

A diagnostic instrument used to assess severity of substance dependence [32]]. The questionnaire consists of 10 items each scored from 1(never) to 4 (always). The LDQ scores are calculated as the total across all items. Higher scores are indicative of greater dependency. (Study 1 α = .82; Study 2 α = .82).

#### Subjective Intoxication Scales (SIS)

SIS consists of six 100mm visual analogue scales used to assess changes in subjective feelings following consumption of alcohol and placebo [33]]. The scales include estimations of light-headedness, irritableness, stimulation, alertness, relaxation and contentedness. (Study 1: α = .83, Study 2: α = .86).

#### Desire for Alcohol Questionnaire- Brief (DAQ)

DAQ assesses current desires/cravings for alcohol [34]]. The DAQ consists of 14 items scored on 7-point Likert scales. While the DAQ was designed to assess different factors of craving (mild desires and intentions, positive and negative reinforcement and strong desires and intentions), previous research has found the factor structure to be inconsistent [35, 36]]. Therefore, mean scores across the scale were used as a measure of craving for both studies. (Study 1: α = .96, Study 2: α = .93).

#### Manipulation check

In order to assess whether the experimental manipulation affected participant’s beliefs regarding their ability to control their drinking , item 3 (“I could easily limit how much I would drink if I drank now”) and 14 (“If I started drinking now I would be able to stop”) on the DAQ scale were used as a manipulation check. Both of these items ask participants to indicate the extent to which they believe they would be able to control their drinking after alcohol. A single variable was created by first reversing both items then summing the scores across these items and dividing by two [35]].

#### Taste test

*Ad lib* alcohol consumption was assessed using an adapted version of the bogus taste test procedure, a widely used and validated method for assessing alcohol intake [37]]. Participants were provided with numbered glasses each containing 225ml of beer. Participants were instructed to rate the beers from 1 to 10 according to five different dimensions, identify the beers alcohol content, brand, and rank the drinks in order of preference. Participants were given 20 minutes to complete this task and were explicitly told to drink as much or as little of the drinks as they pleased. The drinks used in study 1 were; Skol (2.8% ABV) and Skol with 10ml of lemonade.

#### Stop-signal task (SST)

A SST [38]] was run using Inquisit 2.0 (Millisecond Software, Seattle, Washington, 2002) and presented on a 12 inch monitor. Each trial began with the presentation of a white fixation cross (500ms) in the centre of the screen following which an arrow was presented pointing to the right for 50% of trials and to the left for 50% of trials. Participants were required to respond to the stimulus by pressing the corresponding key on the computer keyboard. These stimuli were uninterrupted for 75% of trials but for 25% of trials a stop signal (auditory tone) would be present. Upon hearing the tone participants were required to inhibit responding. The task consisted of four blocks, including a practice block, of 64 trials. Stop signals were presented in a pseudo-randomised order. This version of the task used a tracking algorithm to adjust the delay between the presentation of the ‘go’ stimulus (arrows) and the stop signal (auditory tone). From this task it is possible to assess latency to inhibit responding or stop-signal reaction time (SSRT). SSRT was used as the primary measure of inhibitory control. Go reaction times (reaction times on trials which require a response) and inhibition errors were also analysed.

### Procedure

Testing took place in a semi naturalistic bar laboratory at the University of Liverpool. The bar lab contains items associated with bars, including a stocked fridge, beer pumps, bar stools and seating similar to a typical British pub. At the beginning of both sessions participants provided a breathalyser sample of 0.0mg/l (Lion Alcometer 500, Lion laboratories, Barry, UK) and were assigned to the experimental or control condition.

During the first session participants were weighed and completed a battery of questionnaires (AUDIT, TLFB, LDQ, DAQ and SIS). The second session began with the completion of the DAQ and SIS only. Participants were then administered the priming drinks (alcohol or placebo; order counterbalanced) which they were required to consume within 10 minutes. This was followed by a 10 minute absorption period during which time participants rested.

Following the absorption period, a second breathalyser sample was taken and the DAQ and SIS were completed. Participants were then informed that they would be taking part in a computer task which was designed to assess their ability to exert self-control following the consumption of alcohol. Following completion of the SST, participants were presented with a bogus feedback screen which displayed a ‘self-control index’ score. For participants within the high-control condition, this score was 92.6%, while the score for those within the average-control condition was 51.2%. The experimenter visibly wrote down this score and provided further verbal feedback. Those within the high-control condition were told they were very good at controlling their behaviour following alcohol consumption and were within the top 10% of the population. Conversely, those within the average-control condition were told that they were average at controlling their behaviour following alcohol consumption and that most people scored similarly.

Participants then provided a third breathalyser sample and again completed the DAQ and SIS. At this point participants were also asked to indicate how many units of alcohol they believed the priming drink contained on a 9-point scale (1–9+ units). The taste test was completed and participants provided a final breathalyser sample. Following completion of the second session participants were debriefed and received compensation.

### Data reduction and analysis

In accordance with previous research [39, 40]] reaction time data was trimmed. Reaction times faster than 100ms, slower than 2000ms and more than three standard deviations above the mean were removed. As a number of participants inhibited responding significantly more or less than 50% of the time SSRT was calculated using the integration method [38, 41]]. Participants with negative SSRT’s or those below 50ms were removed from SST analyses. Four participants were removed from SST analysis and due to a technical issue one participant’s SST data was lost. Data for studies 1 and 2 are available on the Open Science Framework (https://osf.io/q3h2z/).

## Study 1: Results

A series of independent sample t-tests revealed no differences between experimental conditions in age, units reported in the TLFB, scores on the LDQ and AUDIT scores (*p*’s≥.301) A chi-square revealed no differences of gender between conditions, χ^2^ (1) = 0.11, *p* = .459 (see Table 1).

**Table 1:** Study 1 participant characteristics for experimental and control group (values mean ±SD).

Characteristic	High-control (*n* = 41)	Average-control (*n* = 40)	Sample (*n* = 81)
Gender (male: female)	23 :18	21:19	44 : 38
Age (years)	23.10 (±4.92)	24.88 (±7.74)	23.98 (±6.49)
Alcohol consumption	45.67 (±28.65)	42.54 (±33.83)	44.12 (±31.16)
AUDIT	11.63 (±4.91)	13.76 (±8.47)	12.69 (±6.94)
LDQ	4.80 (±4.04)	4.85 (±3.79)	4.83 (±3.89)

Alcohol consumption = in UK units (1 unit = 8g alcohol), retrospectively recorded over two weeks. AUDIT = Alcohol Use Disorders Identification Test; scores range from 0(minimum) to 40(maximum). LDQ = Leeds dependence questionnaire, scores range from 0 (minimum) to 30 (maximum).

### Perceived alcohol content

Perceived alcohol content (Table 2) was analysed using a 2x2 mixed ANOVA with a within subject factor of drink (alcohol, placebo) and a between subject factor of condition (high-control/ average-control). There was a main effect of drink, F (1, 79) = 90.19, *p* < .001, η_p_^2^ = .53, with participants estimating there to be significantly more units in the alcoholic drink than in the placebo drink. Perceived alcohol content did not differ between high and average-control conditions and there was no significant drink by condition interaction (*p*’s>.05). A one-sample t-test with a test value of 0 found that participants perceived there to be significantly more than 0 units of alcohol in the placebo drink *t*(80) = 9.43, *p* < .001, *d* = 1.05. This suggests that the placebo was successful and perceived alcohol content did not differ between levels of the between-subject factor.

**Table 2:** Study 1 descriptive statistics for craving, light-headedness, unit estimation and alcohol consumed in the taste test (values mean ± SD).

	Sample						High-control						Average-control					
	Placebo			Alcohol			Placebo			Alcohol			Placebo			Alcohol		
	Baseline	Post-drink	End of session	Baseline	Post-drink	End of session	Baseline	Post-drink	End of session	Baseline	Post-drink	End of session	Baseline	Post-drink	End of session	Baseline	Post-drink	End of session
Light- headed	9.99 (16.29)	18.91 (20.92)	18.53 (17.68)	9.99 (16.29)	41.74 (24.40)	46.70 (24.89)	.12(14.46)	17.05 (18.17)	18.38 (19.35)	10.93 (17.83)	39.24 (23.99)	45.02 (25.31)	.88 (17.76)	22.25 (18.82)	22.70 (17.99)	2.84 (.66)	2.90 (1.99)	49.43 (25.84)
DAQ	2.76(.68)	2.67 (.84)	2.61(.83)	2.94(.67)	3.00 (1.02)	2.91 (1.01)	2.78(.74)	2.65(.83)	2.65(.83)	3.03(.67)	3.09 (1.01)	2.96 (.87)	2.76 (.62)	2.69 (.81)	2.58(.84)	2.84 (.66)	2.90 (1.04)	2.86(1.15)
Unit est	_	_	1.90 (1.81)	_	_	4.14 (1.55)	_	_	1.85 (1.74)	_	_	3.73 (1.41)	_	_	1.90 (1.81)	_	_	4.14 (1.55)
Taste test (ml)	_	_	220.77 (131.44)	_	_	260.37 (129.02)	_	_	222.61 (143.15)	_	_	267.07 (135.88)	_	_	218.88 (120.06)	_	_	253.50 (122.94)

Light-headed scores range from 0(not at all) to 100(extremely). DAQ denotes Desire for Alcohol Questionnaire mean scores range from 1(minimum) to 7 (maximum). Unit est = Unit estimation number of 25ml vodka measures participants believed the priming drink contained from 1 to 9+ (8g of alcohol = 1 UK unit).

### Manipulation check

To investigate whether the manipulation affected perceived ability to control drinking behaviour we used a composite score of DAQ item 3 and 14 which pertain to perceived ability to control drinking. A 2 x 2 x 3 mixed ANOVA was used with condition (high-control, average-control) as a between subject factor and drink (alcohol, placebo) and time (baseline, post-drink, end of session) as a within subject factor. There was a main effect of drink, F (1, 79) = 9.74, *p* = .003, η_p_^2^ = .11, with participants feeling less able to control their drinking during alcohol sessions relative to placebo sessions. There was also a main effect of time, F (1, 158) = 12.72, *p* < .001, η_p_^2^ = .14, with participants reporting an increased ability to control their drinking from post-drink relative to baseline (*p* = .01) and from post-drink to end of session, *p* = .038. However, there were no other main effects of, or interactions with, experimental conditions, suggesting that the manipulation did not affect how well participants believed they could control their drinking (*p*’s >.10).

### Breath alcohol readings (BrAC)

All participants provided a breath alcohol reading of 0.00g/100 ml at the beginning of each session. Following alcohol mean BrAC was 0.31g/100ml (*±*0.10). Following the SST mean BrAC readings significantly decreased to 0.29(*±*0.08), *t* (79) = 2.65, *p* = .010, *d* = 0.27, before significantly increasing, following the taste test, to 0.38(*±* 0.11), *t* (78) = 10.01, *p* < .001, *d* = 0.99. Regarding the placebo session, participants did not consume any alcohol until the taste test, therefore BrAC readings were 0 at baseline and post-drink. Mean BrAC readings following the taste test, within the placebo condition, were 0.07(*±*.06).

### Subjective intoxication

Consistent with previous work [e.g. 17, 18] the light-headedness scale of the SIS was used as the primary measure of subjective intoxication (Table 2). A 2 x 2 x 3 mixed ANOVA with condition (average-control, high-control) as a between-subject variable and drink (alcohol, placebo) and time (baseline, post-drink and end of session) as within-subject variables revealed there to be a significant drink x time interaction, *F* (2, 158) = 75.95, *p* < .001, η_p_^2^ = .49. Least significant difference (LSD) tests revealed that light-headedness significantly increased following alcohol, *p* < .001, and placebo, p < .001. Light-headedness also increased post-drink to end of session within the alcohol session, *p* = .015, but not within the placebo session, *p* = .800. Light-headedness did not differ between alcohol and placebo sessions at baseline, *p* = .122, but was significantly greater following alcohol relative to placebo post-drink, *p* < .001, and at end of session, *p* < .001.

### Craving

A 2 x 2 x 3 mixed ANOVA was used to assess the effect of condition; drink and time on craving (mean DAQ scores; Table 2). The results of this analysis yielded a significant main effect of drink, F (1, 79) = 11.87, *p* = .001, η_p_^2^ = .13, with craving significantly higher during alcohol sessions relative to placebo sessions. There was, however, no main effect of time, F (2, 158) = 1.44, *p =* .240, η_p_^2^ = .02, condition, F (1, 79) = .339, p = .562, η_p_^2^ < .01, and no significant drink x time interaction, F (2, 158) = 1.31, *p =* .273, η_p_^2^ = .02. All other interactions were non-significant (*p’s*>.05).

### Inhibitory control

Three mixed ANOVA’s were used to assess the effect of drink and condition, although we did not expect any effect of condition as participants were exposed to the experimental message following the task, on SST performance (SSRT, inhibition errors and go reaction times). There was no significant main effect of drink, F (1, 74) = 2.69, *p* = .105, η_p_^2^ = .04, condition, F (1, 74) = .599, *p* = .441, η_p_^2^ = .01, and no drink by condition interaction, F (1, 74) = .133, *p* = .133, η_p_^2^ = .03. Similarly, go reaction times were unaffected by drink, F (1, 74) = 3.01, *p* = .087, η_p_^2^ = .04, condition, F (1, 74) = .108, *p* = .744, η_p_^2^ < .01 and the drink by condition interaction, F (1, 74) = .324, *p* = .571, η_p_^2^ < .01.

There was no main effect of drink, F (1, 74) = 1.82, *p* = .182, η_p_^2^ = .02, or condition, F (1, 74) = 3.09, *p* = .083, η_p_^2^ = .04, on inhibition errors. However, there was an unexpected drink by condition interaction, F (1, 74) = 8.05, *p* = .006, η_p_^2^ = .10. This was characterised by greater inhibition errors following alcohol (23.63*±*2.70) relative to placebo (22.38*±*1.96) within the average control condition, *t* (39) = 2.82, *p* = .008, *d* = 0.53, but no difference between alcohol (22.06*±*2.28) and placebo (22.50*±*1.76) in the high control condition, *t* (35) = 1.14, *p* = .264, *d* = 0.22.

We explored this unexpected finding further by running a univariate ANOVA to assess the effect of condition, session and order (which session alcohol and placebo were presented) on inhibition errors. A main effect of condition, F (1, 144) = 4.03, *p* = .047, η_p_^2^ = .03 was found and was superseded by a significant condition by session by order interaction, F (1, 144) = 5.09, *p* = .026, η_p_^2^ = .03. An LSD test revealed the three-way interaction to be the result of significantly greater inhibition errors when consuming alcohol (23.90 *±* 1.97) relative to placebo (21.90 ± 1.58) but only when alcohol was consumed in the second session and only within the average-control condition, *p* = .004. There were no other significant differences, main effects or interactions (*p*’s >.05).

This finding suggests that SST performance in the second session was affected by the bogus feedback provided to them in the first session. Specifically, participants who were led to believe they had average self-control in the previous session performed worse in the second session when consuming alcohol.

### Taste test

A final mixed ANOVA was used to assess the effect of condition and drink on amount of beer consumed (in ml) during the taste test (Table 2). There was a significant main effect of drink, F (1, 79) = 7.74, *p* = .007, η_p_^2^ = .09, with participants consuming significantly more alcohol within the alcohol condition relative to the placebo condition. There was no significant drink x condition interaction, F (1, 79) = .12, *p* = .730, η_p_^2^ < .01, and no main effect of condition, F (1, 79) = .116, *p* = .734, η_p_^2^ < .01.

## Interim discussion

In study 1 participants were provided with bogus feedback following a SST which suggested that their ability to control their behaviour was high or average. It was predicted that the alcohol priming effect would be mitigated when participants were led to believe that their ability to self-regulate was high following alcohol. The findings of study 1 do not support this hypothesis. Results suggest that an alcohol priming effect occurred as participants consumed more alcohol during the taste test following a priming dose of alcohol relative to placebo and that this occurred in the absence of alcohol-induced impairments in inhibitory control. However, while craving was higher overall during the alcohol session, craving did not further increase following the alcohol prime. Contrary to previous findings [24, 25]], there was no difference between participants led to believe they had high levels of self-control and those led to believe they had average self-control on any measure of alcohol seeking. We also found that performance on the SST in the second session was affected by bogus feedback provided to them in the first session. Those led to believe their self-control was average had higher rates of inhibitory errors in the second session but only when alcohol was consumed. Study 2 was designed to test the effect of such beliefs on SST performance by manipulating beliefs prior to a SST.

In study 1 it was implicitly suggested that self-control following an acute dose of alcohol may affect subsequent drinking behaviour as participants were led to believe that their behavioural regulation was high or average following alcohol consumption. However, this manipulation did not affect how well participants believe they are able to control their drinking, suggesting that the manipulation was not successful. This manipulation may have been too subtle in order to affect beliefs about the effects of alcohol on control over drinking. Therefore study 2 addressed this by using a more explicit manipulation in which participants were directly told that consuming an acute dose of alcohol would reduce the urge to drink.

## Study 2: Method

In study 2, we exposed participants to an experimental script which explicitly stated [see 22, 23]] that consuming a dose of alcohol would *reduce* the urge to drink or were provided with a neutral control message. Participants were exposed to this script prior to a priming drink and SST. It was hypothesised that the alcohol priming effect and alcohol-induced inhibitory impairments would be reduced following the experimental message.

### Participants

Eighty-two participants (29 male, 53 female) aged 18–48 (*M* = 26.30, *SD* = 8.01) were recruited via advertisements placed around the University of Liverpool or in return for course credit. Inclusion and exclusion criteria were identical to study 1.

### Materials

In order to vary the taste tests which are used within the department the taste test within this study used three drinks Carlsberg (3.8% ABV), Becks Blue (< .05% ABV) and Fosters (4.0% ABV). All other materials were identical to the first study.

### Procedure

The procedure for study 2 matched study 1. The only difference being that prior to the priming drink, the experimenter exposed participants to one of two messages under the pretence that they were informing the participant of the findings of the research programme so far. The experimental group were exposed to a message which suggested that consuming a small dose of alcohol would actually reduce the urge to drink:

Our research has found that consuming alcohol reduces the body’s urge to drink as the body quickly becomes sated once it has received a small dose of alcohol, reducing the biological urge to drink. Furthermore, we have found that consuming large amounts of alcohol as part of an unplanned binge is a cultural phenomenon found in the UK and Ireland. Other European countries involved in our research program have not found that consuming alcohol leads to further alcohol consumption.

Meanwhile, the control group were provided with a control message:

Our research has been investigating the effects of alcohol on thought processes like memory, problem solving and attention. We have so far found that alcohol has a greater effect on some of these processes than others. This final experiment is testing the effects of alcohol on simple reaction times and taste perception.

Within the second session participants were reminded of this information. The participants then completed the same procedure as study 1 (without bogus feedback following the SST) and were asked at the end of the second session to complete a two week alcohol diary to assess whether the experimental manipulation was successful outside of the lab (see S1 Text). Participants were fully debriefed when they returned with the diary.

### Data reduction and analysis

Data reduction and analysis was the same as for the first study. SST data from two participants in the control group were lost due to technical issues. An additional 6 participants presented SSRT’s below 50ms and so were not included in SST analysis.

## Study 2: Results

Independent samples t-tests revealed no difference between the two script conditions for age, TLFB, AUDIT and LDQ scores (all *p*’s≥.551). A chi square revealed no gender differences between conditions, χ ^2^ (1) = .01, *p* = .565 (Table 3).

**Table 3:** Study 2 participant characteristics for experimental and control conditions (values mean ±SD).

Characteristic	Experimental (*n* = 42)	Control (*n* = 40)	Sample (*n* = 82)
Gender (male: female)	14:27	14:26	29:53
Age (years)	26.66 (±8.16)	25.86 (±7.95)	26.30 (±8.01)
Alcohol consumption	45.43 (±18.91)	45.15 (±17.29)	45.29 (±18.02)
AUDIT	11.54 (±4.07)	12.13 (±4.75)	11.83 (±4.40)
LDQ	4.54 (±4.15)	5.08 (±4.00)	4.80 (±4.06)

Alcohol consumption = in UK units (1 unit = 8g alcohol), retrospectively recorded over two weeks. AUDIT = Alcohol Use Disorders Identification Test; scores range from 0(minimum) to 40(maximum). LDQ = Leeds dependence questionnaire, scores range from 0 (minimum) to 30 (maximum).

### Perceived alcohol content

There was a significant main effect of drink on the amount of alcohol perceived to be in the priming drink, F (1, 79) = 163.20, *p* < .001, η_p_^2^ = .67, with participants estimating there to be significantly more units of alcohol in the alcoholic drink than in the placebo (Table 4). There was no significant drink x script interaction and no main effect of script (*p’s*>.05). A one-sample t-test found that participants estimated there to be significantly more than 0 units in the placebo drink, *t*(79) = 11.24, *p* < .001, *d* = 1.26.

**Table 4:** Study 2 descriptive statistics for craving, light-headedness, unit estimation and alcohol consumed in the taste test (values mean ± SD).

	Sample						Experimental						Control					
	Placebo			Alcohol			Placebo			Alcohol			Placebo			Alcohol		
	Baseline	Post-drink	End of session	Baseline	Post-drink	End of session	Baseline	Post-drink	End of session	Baseline	Post-drink	End of session	Baseline	Post-drink	End of session	Baseline	Post-drink	End of session
Light- headed	9.95 (16.16)	19.62 (18.57)	20.49 (18.71)	4.61(8.19)	40.88 (22.51)	47.39 (25.48)	8.12(14.46)	17.05 (18.17)	18.38 (19.35)	4.14 (8.92)	41.90 (22.24)	45.36 (25.27)	11.88 (17.76)	22.25 (18.82)	22.70 (17.99)	5.07 (7.48)	39.90 (22.99)	49.43 (25.84)
DAQ	2.52(.83)	2.65 (.88)	2.63(.92)	2.63(.78)	2.87 (.91)	2.88 (.99)	2.57(.94)	2.60(.83)	2.67(.96)	2.56(.78)	2.78 (.92)	2.83 (1.01)	2.47 (.70)	2.70 (.95)	2.59(.90)	2.47 (.70)	2.70 (.95)	2.92(.90)
Unit est	_	_	1.40 (1.12)	_	_	4.06 (1.54)	_	_	1.29 (1.04)	_	_	4.17 (1.65)	_	_	1.53 (1.20)	_	_	3.95 (1.43)
Taste test (ml)	_	_	292.48 (182.33)	_	_	298.61 (179.47)	_	_	298.61 (183.09)	_	_	295.70 (178.20)	_	_	286.20 (183.66)	_	_	301.66 (182.96)

### Manipulation check

There was a main effect of drink which was superseded by a significant drink x script interaction on the manipulation check, F (1, 77) = 5.53, *p* = .021, η_p_^2^ = .07. Within the control condition, participants reported feeling less able to control their drinking during the alcohol session relative to the placebo session, *p* < .001. However, reported ability to control drinking did not differ between alcohol and placebo sessions within the experimental condition, *p* = .462. This suggests the experimental message was successful in reducing the belief that alcohol would lead to a loss of control over drinking.

### Breath alcohol readings (BrAC)

Following alcohol mean BrAC reading was 0.30 g/100ml (*±* 0.15). Following completion of the SST, mean BrAC readings significantly decreased to 0.26 (*±*0.10), *t* (76) = 2.84, *p* = .006, *d* = 0.32, before increasing significantly to 0.36 (*±*0.15) following the taste task, *t* (72) = 7.63, *p* < .001, *d =* 0.78. Participants did not consume alcohol until the taste test within the placebo sessionFollowing the taste test mean BrAC readings were 0.09 (*±*0.10).

### Subjective intoxication

There was a significant drink x time interaction, F (2, 144) = 73.68, *p* < .001, η_p_^2^ = .49, with light-headedness increasing from baseline to post-drink, *p* < .001, and from post-drink to end of session, *p* = .001, following alcohol (Table 4). Light-headedness also increased following placebo from baseline to post-drink, *p* < .001, and from baseline to end of session, *p* < .001, but not from post-drink to end of session, *p* = .372. There was an unexpected significant difference between alcohol and placebo conditions in baseline light-headedness, *p* = .001, with participant’s reporting higher baseline light-headedness prior to placebo administration relative to alcohol. However, light-headedness was greater within the alcohol condition post-drink, *p* < .001, and at end of session, *p* < .001.

### Craving

There was a significant main effect of drink, F (1, 77) = 13.63, *p* < .001, η_p_^2^ = .15, and time, F (2, 154) = 6.30, *p* = .002, η_p_^2^ = .08, on craving (Table 4). However, there was no drink x time interaction, F (2, 154) = 1.01, *p* = .366, η_p_^2^ = .01, or main effect of script, F (1, 77) = .087, *p* = .769, η_p_^2^ < .01. Overall, participants reported higher levels of craving within the alcohol condition than within the placebo condition. During both drink sessions, craving increased from baseline to post-drink, *p* = .009, and from baseline to end of session, *p* = .003, but not from post-drink to end of session, *p* = .943.

### Inhibitory control

**Fig 1:**
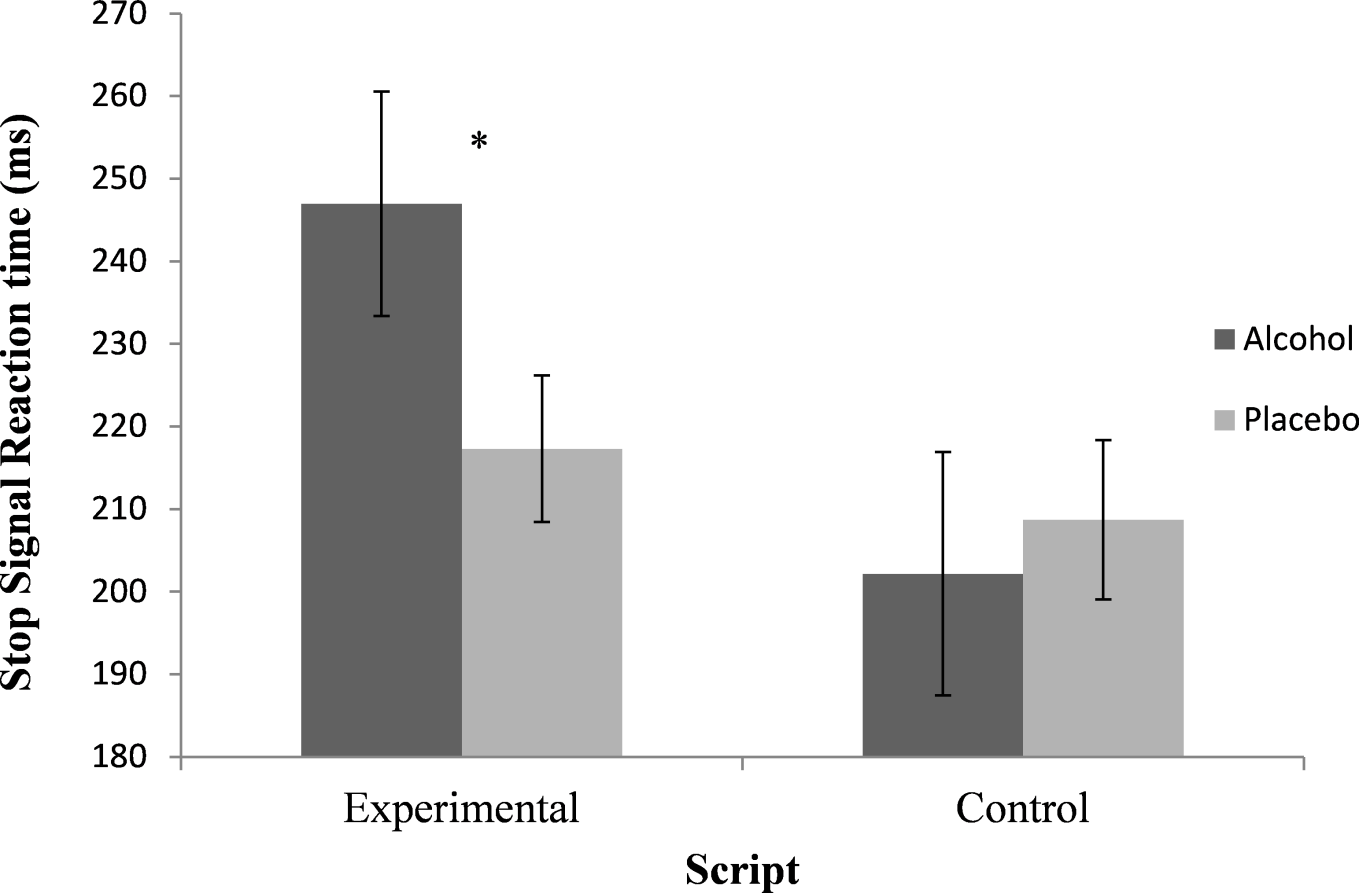
Mean integrated SSRT’s (ms) following alcohol and placebo for both the experimental and control condition. Values are mean *±* SEM, *script compared to control condition (*p = .012).

There was no significant effect of drink on SSRT, F (1, 72) = 2.45, *p* = .122, η_p_^2^ = .03. There was also no significant main effect of script, F (1, 72) = 3.07, p = .084, η_p_^2^ = .04. However, there was a significant drink x script interaction, F (1, 72) = 6.00, *p* = .017, η_p_^2^ = .08. This interaction was the result of participants having greater SSRT’s following alcohol (246.96*±* 108.58), relative to placebo (217.31*±*68.89), within the experimental condition, *t* (39) = 2.65, *p* = .012, *d* = 0.33, but there being no difference in SSRT’s between alcohol (202.18 *±*46.89) and placebo (208.71 ±35.58) session within the control condition, *t* (33) = .71, *p* = .480, *d* = 0.16 (see Fig 1).

There was a significant main effect of drink on inhibition errors, F (1, 72) = 8.22, *p* = .005, η_p_^2^ = .10, with a higher error rates within the alcohol (23.85 *±*3.29) relative to placebo condition (23.09*±*2.47). There was no significant drink x script interaction or main effect of script on inhibitory failures. There was also no significant main effect of drink, script and no significant drink x script interaction on go reaction times (*p*’s >.05). Overall, this indicates that the pharmacological effects of alcohol led to increased failures to inhibit responding. However, alcohol-induced impairments of SSRT were only present when participants were led to believe alcohol would reduce their urge to drink.

### Taste test

There was no significant main effect of drink, F (1, 78) = .066, *p* = .798, η_p_^2^ < .01, on the amount of alcohol consumed in the bogus taste test. In addition, there was no significant drink x script interaction, F (1, 78) = .801, *p* = .373, η_p_^2^ = .01, and no significant main effect of script, F (1, 78) = .002, *p* = .963, η_p_^2^ < .01.

## General discussion

The current research aimed to manipulate beliefs about the effects of alcohol on behavioural regulation and assess the effect of such beliefs on the alcohol priming effect. In addition, study 2 also explored the extent to which beliefs about the effects of alcohol can influence alcohol-induced impairments in inhibitory control. Study 1 provided participants with bogus feedback following a SST suggesting that they had high or average levels of self-control following alcohol. This study found no effect of this manipulation on alcohol consumption or craving; although it did reveal an alcohol priming effect with alcohol-induced increase in *ad lib* consumption and higher levels of craving within alcohol sessions. It also suggested that performance on the SST was affected by bogus feedback they had received in the previous session.

In study 2 participants were either explicitly told that small doses of alcohol *reduce* the urge to drink or were provided with a control message. This occurred prior to receiving a priming drink and completing the SST. It was hypothesised that the alcohol priming effect and inhibitory control impairments would be reduced among those led to believe that a small dose of alcohol would reduce their urge to drink. As with the first study, the current findings do not support this hypothesis. While craving increased over time regardless of which drink was consumed, craving was higher in the alcohol session, relative to the placebo session and *ad lib* alcohol consumption did not differ between sessions. There was no effect of script on *ad lib* consumption or craving. While there was no difference between alcohol and placebo session on go reaction times, inhibition errors were greater following alcohol. Importantly, following exposure to the experimental script there was evidence of alcohol-induced impairments of SSRT (greater SSRT following alcohol, relative to placebo) but no impairments were present following the control message.

Neither study 1 or 2 found beliefs regarding the effects of alcohol to affect alcohol-seeking. While this contradicts previous work which has suggested high perceived levels of behavioural regulation to be associated with increased substance use relative to low levels [24, 25]], it is important to note that the manipulation check suggests study 1’s manipulation was unsuccessful. Alternatively, previous findings may have been driven by participants who, led to believe that their ability to control their behaviour is low, reduce their substance use. Indeed, the current studies did not employ a low-control condition (study 1) or a condition suggesting alcohol will *increase* the urge to drink (study 2). In future, these conditions should be employed to properly disentangle the effects of perceived ability to self-regulate on subsequent drinking.

In study 2 participants who were informed alcohol would reduce the urge to drink had poorer inhibitory control following alcohol, than control participants. This may occur as participants infer from the message that alcohol will not impair their ability to control their behaviour. Therefore any compensatory effects which would usually occur, in an attempt to overcome alcohol’s impairing effects, are not present. Indeed, Fillmore et al [20]] found that participants led to expect alcohol-induced impairment on a pursuit rotor task performed better than participants led to expect improvement following alcohol. The authors suggest this is a result of participants attempting to compensate for the expected impairing effects of alcohol. Importantly, ours is the first study to suggest that the expectation that alcohol will not impair self-regulation can lead to poorer SSRT’s. Conversely, inhibition errors were increased following alcohol relative to placebo regardless of belief. In study 1 we found that those led to believe they had average self-control had higher rates of inhibitory errors in the second session when alcohol was consumed. This is in contrast to study 2’s finding that performance worsened when participants believed their ability to self-regulate would not be impaired by alcohol. Study 1 was, however, not designed to assess the effect of beliefs on SST performance. Moreover, the manipulation in study 1 did not affect beliefs about the ability to control behaviour following alcohol but may have affected general beliefs about self-regulation. The effect of alcohol on inhibitory control may, therefore, be partly explained by individual differences in beliefs about the effects of alcohol [18]]. Future studies should take into account participant’s beliefs about the effects of alcohol on their ability to control their behaviour when assessing the effect of alcohol on inhibitory control.

Study 1 replicated previous research, showing that initial alcohol consumption can increase (prime) alcohol-seeking [2–5, 16, 17]]. However, *ad lib* beer consumption did not differ between alcohol and placebo sessions within study 2, although, craving was higher in the alcohol sessions. While study one found that more alcohol was consumed in the taste test following alcohol there was no evidence of inhibitory control impairments. In contrast, study 2 found evidence of inhibitory control impairments following an acute dose of alcohol but no evidence of an alcohol priming effect with evidence of increased craving following alcohol and placebo. This contradicts previous suggestions that such impairments mediate subsequent alcohol consumption [6, 11]] and supports research which has not found an association between inhibitory impairments and the alcohol priming effect [4, 12–15]].

There are a number of limitations with the current studies. Firstly, the manipulation in study 1 did not affect participant’s beliefs regarding their ability to control drinking and so was not successful. We exposed participants to a ‘self-control index’ score following completion of the stop-signal task. Previous studies [24, 25]] have also included additional cognitive tasks, ostensibly to measures participants’ behavioural regulation, and exposed participants to further information about their ‘scores’. These components may increase the believability of the manipulation. Secondly, neither study contained a baseline measure of SST performance (i.e. before the priming drink) to account for individual differences in performance, although repeated testing using such tasks has been demonstrated to be problematic (see [42]). Thirdly, while craving was higher in alcohol sessions for both studies this was an overall difference and so may not have been increased following consumption of the priming drink. Furthermore, we did not provide participants with water following the priming dose. It is therefore possible that alcohol residue in the mouth may have inflated BrAC readings. However, readings are similar to previous work which has used comparable doses [43]]. Finally, both studies used different versions of the bogus taste test and so the amount of alcohol consumed between the two studies may not be directly comparable. While this is not ideal this was done in order to vary taste tests which are used in the department. Importantly, while the drinks differed, the form of the taste test remained the same and a number of different versions of the bogus taste test have been found to be valid [37]].

In summary, neither of the two studies found that beliefs regarding alcohol’s ability to control behaviour, following beverage consumption, moderated the alcohol priming effect. However, this may be due to the absence of a low control group, for study 1, or a group led to believe an acute dose of alcohol will *increase* the urge to drink, in the case of study 2. This research adds to a growing body of research that suggests impairments in inhibitory control do not contribute to the alcohol priming effect. It is also the first to suggest that alcohol-induced impairments of inhibitory control may be influenced by beliefs about the effects of alcohol. Future studies should investigate the role of beliefs about the effects of alcohol on individual differences in alcohol-induced inhibitory control impairments and the potential effect of these beliefs on other widely used measures of inhibitory control.

## Supporting information

S1 Text Alcohol diary results. (DOCX)
