# Supplementary material for: The effect of beliefs about alcohol’s acute effects on alcohol priming and alcohol-induced impairments of inhibitory control
Source: PLoS One. 2018 Jul 26;13(7):e0201042. doi: 10.1371/journal.pone.0201042 (PMC6062075; doi:10.1371/journal.pone.0201042)
Supplement: S1 Text — (DOCX) [file pone.0201042.s001.docx]

**Supporting Information**

**S1text. Alcohol diary results**

Sixty seven participants returned the alcohol diaries in total (36 in the experimental condition, 31 in the control). A between subjects t-test revealed there to be no significant differences between the control and experimental condition on the amount of alcohol consumed 2 weeks following the second testing session, *t* (65) =0.27, *p*=.787, *d*=0.07.
